# Thoracotomy for emergency repair of iatrogenic tracheal rupture: single center analysis of perioperative management and outcomes

**DOI:** 10.1186/s12871-019-0869-5

**Published:** 2019-10-27

**Authors:** Manuel F. Struck, Gunther Hempel, Uta C. Pietsch, Johannes Broschewitz, Uwe Eichfeld, Robert Werdehausen, Sebastian Krämer

**Affiliations:** 1 Department of Anesthesiology and Intensive Care Medicine, University Hospital Leipzig, Liebigstr.20, 04103 Leipzig, Germany; 2 Department of Visceral, Transplantation, Thoracic and Vascular Surgery, University Hospital Leipzig, Liebigstr. 20, 04103 Leipzig, Germany

**Keywords:** Iatrogenic tracheal rupture, Surgical repair, Thoracotomy, Perioperative management, Anesthesia, Airway management, One-lung ventilation, Complication

## Abstract

**Background:**

Iatrogenic tracheal ruptures are rare but life-threatening airway complications that often require surgical repair. Data on perioperative vital functions and anesthetic regimes are scarce. The goal of this study was to explore comorbidity, perioperative management, complications and outcomes of patients undergoing thoracotomy for surgical repair.

**Methods:**

We retrospectively evaluated adult patients who required right thoracotomy for emergency surgical repair of iatrogenic posterior tracheal ruptures and were admitted to a university hospital over a 15-year period (2004–2018). The analyses included demographic, diagnostic, management and outcome data on preinjury morbidity and perioperative complications.

**Results:**

Thirty-five patients who met the inclusion criteria were analyzed. All but two patients (96%) presented with critical underlying diseases and/or emergency tracheal intubations. The median time (interquartile range) from diagnosis to surgery was 0.3 (0.2–1.0) days. The durations of anesthesia, surgery and one-lung ventilation (OLV) were 172 (128–261) min, 100 (68–162) min, and 52 (40–99) min, respectively. The primary airway management approach to OLV was successful in only 12 patients (34%). Major complications during surgery were observed in 10 patients (29%). Four patients (11%) required cardiopulmonary resuscitation, one of whom received extracorporeal membrane oxygenation, and another one of these patients died during surgery. Major complications were associated with significantly higher all-cause 30-day mortality (*p* = 0.002) and adjusted mortality (*p* = 0.001) compared to patients with minor or no complications.

**Conclusions:**

Surgical repair of iatrogenic tracheal ruptures requires advanced perioperative care in a specialized center due to high morbidity and potential complications. Airway management should include early anticipation of alternative OLV approaches to provide acceptable conditions for surgery.

## Background

Iatrogenic tracheal ruptures of the posterior membrane are rare but life-threatening airway complications that are associated with emergency tracheal intubation, tracheotomy, and surgery, and blunt and penetrating trauma [1]. The literature gives only rough estimations of its prevalence and incidence and current knowledge results mainly from retrospective studies and case series. Tracheal intubation-related incidence is reported to be approximately 0.005% for single lumen intubations and may be between 0.05 and 0.19% for double lumen intubations, while the incidence of iatrogenic tracheal ruptures caused by percutaneous dilation tracheostomies may be as high as 1% [1–11].

Although conservative and less invasive treatment options have become increasingly popular, larger lesions associated with severe respiratory impairment still require direct surgical repair [1–8]. Surgical approaches from the anterior neck are possible in lesions of the cervical part of the trachea, whereas right thoracotomy under one-lung ventilation (OLV) is the method of choice for lesions of the thoracic trachea, tracheal bifurcation and proximal right main bronchus [1–11]. Surgery includes suture of the lesion, sealing and reinforcement of the sutures [9–11]. Depending on the anatomy and extension of the lesion, perioperative airway management and OLV may require frequent fiber-optic re-evaluation of the tube position to provide the best conditions for surgery.

In the literature, the occurrence of clinically relevant issues (e.g., impaired view of the operating field, cardiopulmonary deterioration, or bleeding) during emergency tracheal surgery is not sufficiently examined, and studies that focus particularly on the course of perioperative vital functions are not available. The goal of this study was to explore perioperative management, complications during surgical repair, and outcomes of iatrogenic tracheal ruptures based on individual comorbidity. Main study endpoint was to detect, whether major perioperative complications may influence patient outcomes compared to none or minor perioperative complications. Based on observed results, we tried to formulate a clinical message and practice implications for the treatment of patients undergoing thoracotomy for surgical repair of iatrogenic tracheal ruptures.

## Methods

### Study design and ethics

We conducted a retrospective single-center cohort study on patients who required thoracotomy for surgical repair of iatrogenic tracheal ruptures. After approval of the Ethics Committee, the database of the University Hospital Leipzig was reviewed to identify patients who were classified by the ICD-10 system for tracheal rupture (ICD-10 code S11.x and S27.x) between 07/2004 and 12/2018. Iatrogenic tracheal rupture was defined as a lesion of the posterior part of the trachea caused by tracheal intubation, tracheotomy, surgery, and blunt and penetrating trauma. Patients with incomplete documentation, anterior tracheal injury, cervical surgical approach without OLV, conservative approach and age under 18 years old were excluded. The study protocol adheres to the STROBE guidelines for uniform reporting of observational studies. The University Hospital Leipzig is a 1350-beds academic medical center that provides advanced emergency care and a referral center for acute respiratory distress syndrome (ARDS) and extracorporeal membrane oxygenation (ECMO). All interdisciplinary specialists for the treatment of tracheal ruptures are available 24/7, i.e., anesthetists and critical care specialists, endoscopy teams (pulmonologists and gastroenterologists), radiologists, otolaryngologists, and thoracic and upper gastrointestinal surgeons.

### General management

Patients with suspected iatrogenic tracheal rupture were referred by emergency medical service (EMS) transport from other hospitals or were already admitted to the intensive care unit (ICU), the normal ward or the operating room of the university hospital. After diagnostic confirmation using fiber-optic bronchoscopy and chest computed tomography (CT), all patients were admitted (or had already been admitted) to the ICU. The decision for surgery was made by a team approach including attending thoracic surgeons, pulmonologists and critical care physicians according to patients’ condition, rupture size, injury mechanism and diagnostic findings (e.g., mediastinitis, pneumothorax, pleural effusion). Emergency surgery was scheduled, and the patients were transferred to the operating room and underwent thoracotomy for surgical repair following general anesthesia and one-lung ventilation. Patients were returned to the ICU postoperatively.

Patients were analyzed for demographic data, including age, body mass index, morbidity (American Society of Anesthesiologists, ASA, classification), sequential organ failure assessment (SOFA) and simplified acute physiology score revision two (SAPS II) before tracheal rupture, size and anatomy of the lesion, anesthesia regimen, airway management (e.g., tube advancement into one main-stem bronchus, double-lumen tube (DLT), bronchus blocker), surgical procedures, suture reinforcement techniques, process times of anesthesia, surgery and OLV, paO_2_/FiO_2_-ratios (p/f) and serum lactate levels before and after surgery, proportion of pure oxygen ventilation, peak inspiratory pressures (PIP) and positive end expiratory pressures (PEEP), lowest SpO_2_ levels, highest end-tidal CO_2_ levels, lowest systolic blood pressure (SBP), highest noradrenaline dosage, adrenaline and dobutamine administration, fluid volumes, transfusion amount, total blood loss, urinary output, length of stay in the ICU (LOS ICU), ventilator days after surgery, and all-cause and adjusted 30-day mortality.

Perioperative complications were analyzed and classified as none, minor and major complications. Minor complications were defined as the occurrence of at least one of the following criteria: one necessary change of airway management to establish sufficient OLV, mild to moderately impaired visualization of the situs, noradrenaline dosage > 0 and ≤ 0.5 μg kg min^− 1^, adrenaline dosage > 0 and < 1 mg, oliguria and anuria, and short-term hypotension (SBP < 90 mmHg), hypoxemia (SpO_2_ < 90%) and hypercapnia (etCO_2_ > 50 mmHg). Major complications were defined as cardiac arrest, noradrenaline dosage > 0.5 μg kg min^− 1^, adrenaline dosage ≥1.0 mg, transfusion of greater than four units of red blood cells (RBC), fresh frozen plasma (FFP) or platelets in total, more than one required change of airway management to achieve sufficient OLV, severely impaired visualization of the situs, and sustained hypotension, hypercapnia or hypoxemia despite documented efforts of treatment.

Data from perioperative management were obtained electronically, including automatic transfer of monitored vital functions (heart rate, SpO_2_, etCO_2_, arterial blood pressure, central venous pressure, body temperature via urinary bladder sensor or rectal probe) and anesthesia unit (FiO_2_, inspiratory and end-expiratory anesthesia gas concentrations, minute volumes) using the electronic COPRA patient data management system (COPRA system GmbH, Berlin, Germany). Due to automatic electronic data transfer, a reliable continuous data collection without missing data was guaranteed in the presented data. Additional electronic or paper-based documentation was included in the analysis if present.

### Statistical analysis

Data are reported as medians (interquartile range, IQR), and counts (percentage), unless indicated otherwise. Due to heterogeneity of patients, unspecific underlying causes, and rare and unpredictable emergency conditions, a precise sample size calculation was not possible for this study but we expected a patient number of at least two patients per analyzed year, according to previous studies of our and other centers [1–11]. Statistical comparisons between patients with and without major perioperative complications were performed using the χ^2^ test and Fisher’s exact test for qualitative data, and the Mann-Whitney U-test was used for quantitative data. The alpha level of significance was set at 0.05. All tests were two-tailed. Multivariate analysis was not performed due to the small sample size. Statistical analyses were performed using IBM SPSS Statistics for Windows, version 24.0 (IBM Corp., Armonk, NY, United States).

## Results

**Fig. 1:**
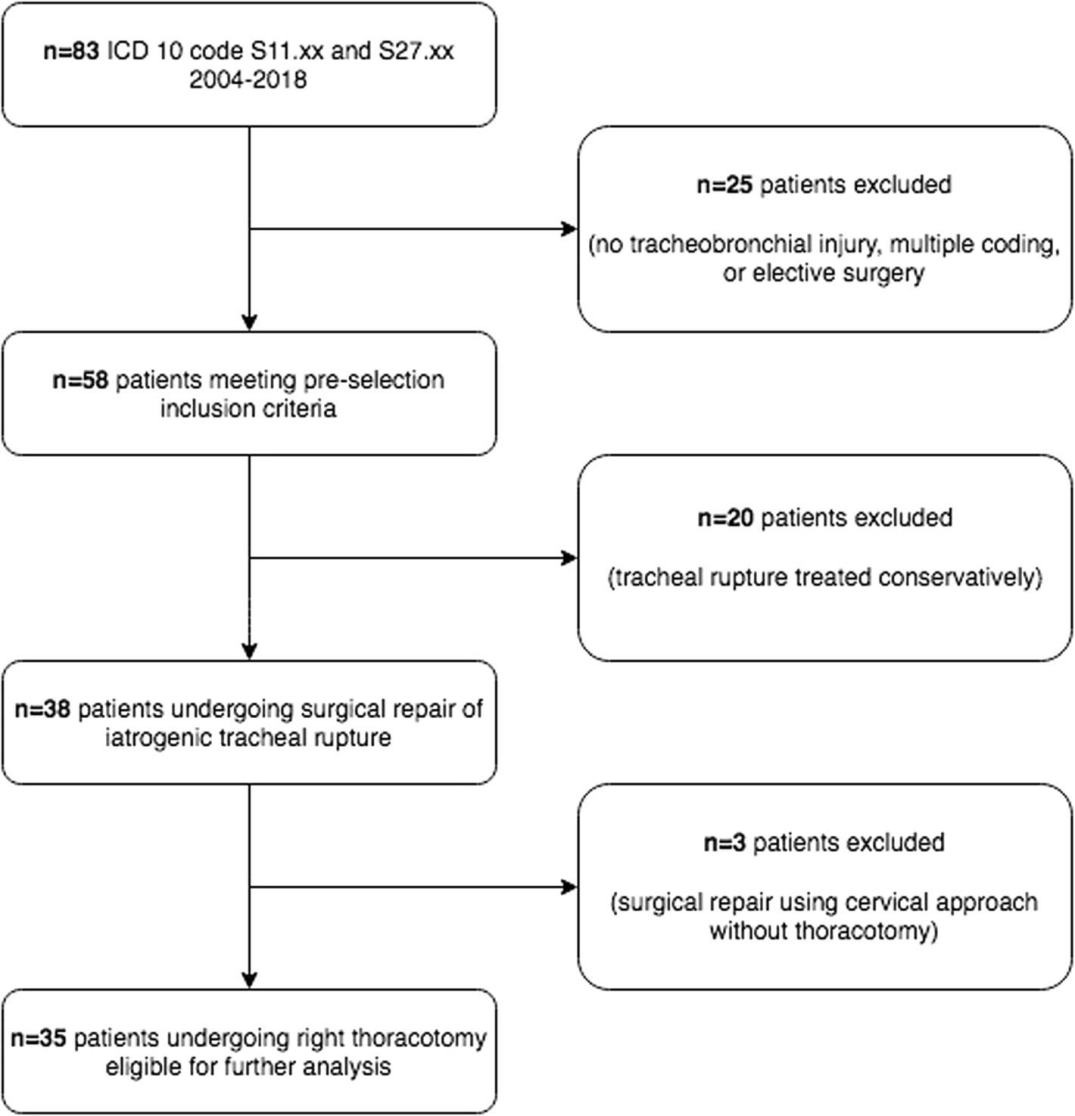
Study flow chart

**Fig. 2:**
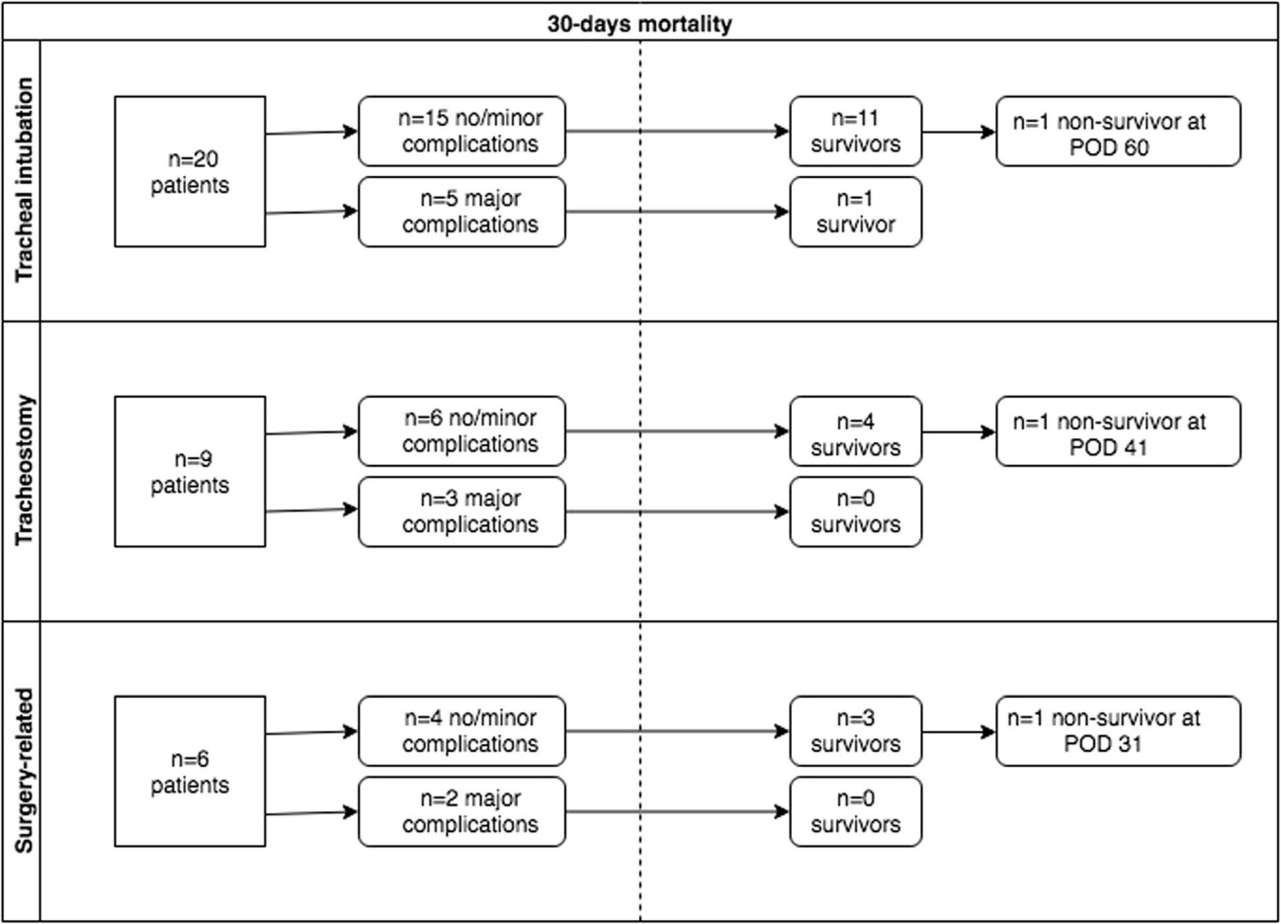
Linear flow chart of different causes of iatrogenic tracheal ruptures. POD: post-operative day

During the study period, 83 patients were identified according to their classification as ICD-10 code S11.x (open wounds to the trachea) and S27.x (open wounds to the thoracic trachea). Twenty-five patients were excluded due to absence of tracheobronchial injury, multiple coding or elective surgery. Of the remaining 58 patients, 20 patients had tracheobronchial rupture treated conservatively (with or without stent therapy) and three underwent minimal invasive surgery (anterior approach) without thoracotomy. Thirty-five patients met the inclusion criteria and were subject of the study (Figs. 1 and 2).

### Patients’ characteristics

**Fig. 3:**
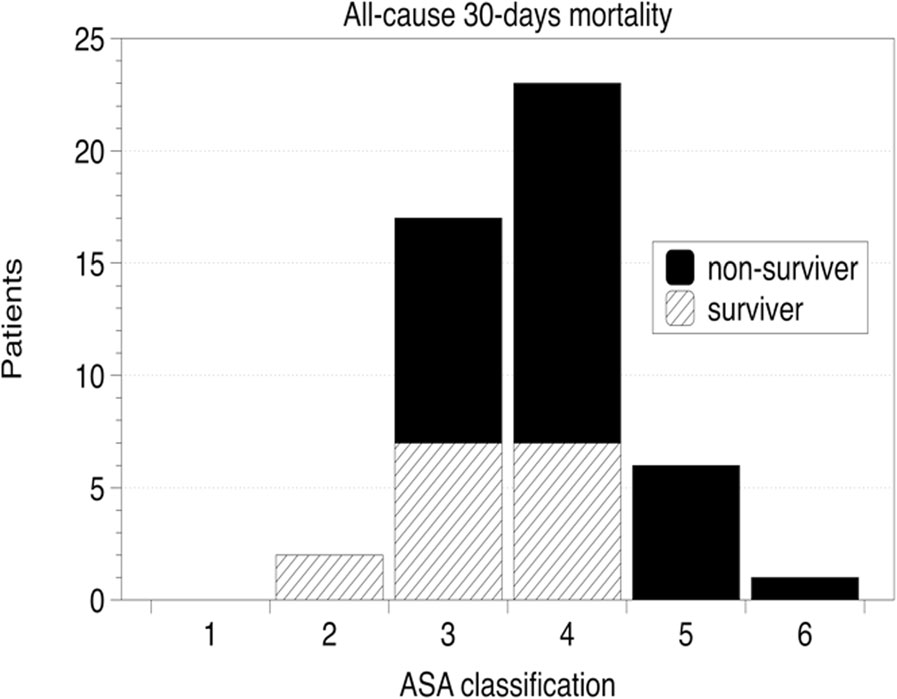
American Society of Anesthesiologists (ASA) classification and all-cause 30-day mortality of patients with iatrogenic tracheal ruptures and surgical repair

There were 25 female patients (71%) and 10 male patients (29%) with a median age of 67 years (range 26–86 years). One-half of the patients (*n* = 17, 49%) were referred from other hospitals. All but two patients (96%) presented with critical conditions (ASA III-VI) prior to iatrogenic tracheal rupture, including one patient who required extracorporeal lung assist (ECLA) due to severe hypercapnia (Fig. 3). Nineteen patients (54%) were already admitted to the ICU prior iatrogenic tracheal rupture and seven patients (20%) underwent emergency tracheal intubation due to cardiopulmonary resuscitation (CPR). The main cause of tracheal rupture was tracheal intubation (*n* = 20, 57%), followed by tracheotomy-related causes (*n* = 9, 26%) (four percutaneous dilatation tracheotomy (PDT), two surgical tracheotomy, two lesions associated with tracheal cannula reinsertion, and one during bronchoscopy), and surgical injuries (*n* = 6, 17%) (three patients each with esophageal and otolaryngeal surgery) (Fig. 2). Subcutaneous emphysema as a primary symptom was present in 27 patients (77%), and three patients (9%) primarily presented with signs of mediastinitis, with two of these occurrences after esophageal surgery and one occurrence after emergency tracheal intubation due to severe exacerbation of chronic obstructive pulmonary disease and delayed referral. Further data on demography and comorbidity are presented in Table 1.

**Table 1:** Demography, clinical presentation, perioperative care, and outcomes of patients with iatrogenic tracheal rupture

	Total (*n* = 35)	No and minor complications (*n* = 25)	Major complications (*n* = 10)	*P*
Age, years	67 (55–76)	61 (53–73)	75 (58–82)	0.125
Female	25 (71)	18 (72)	7 (70)	0.100
BMI Clinical presentation	26 (24–31)	27 (27–32)	24 (21–29)	0.213
ASA	4 (3–4)	4.0 (3–4)	4.0 (3.75–5)	0.313
ICU before rupture	19 (54.3)	14 (56)	5 (50)	0.100
CPR before rupture	7 (20)	5 (20)	2 (20)	0.100
SAPS II	52 (36–58)	49 (33–58)	56 (49–67)	0.339
SOFA	7 (4–9)	7 (4–9)	6 (5–9)	0.567
Tear length, cm	5 (4–6)	5 (4–6)	4 (4–6)	0.737
Causative events				0.438
Tracheal intubation	20 (57)	15 (60)	5 (50)	
Emergency intubation	18 (51)	13 (52)	5 (50)	
Tracheotomy	9 (26)	7 (28)	2 (20)	
Surgery	6 (17)	3 (12)	3 (30)	
Interfacility EMS referral	17 (49)	11 (44)	6 (60)	0.392
Process times				
Rupture to surgery, days	0.3 (0.2–1.0)	0.3 (0.2–0.8)	0.7 (0.2–3.4)	0.272
Anesthesia, minutes	172 (128–261)	160 (125–209)	243 (149–304)	0.093
Surgery, minutes	100 (68–162)	97 (64–121)	141 (78–222)	0.265
OLV, minutes	52 (40–99)	55 (40–91)	63 (46–109)	0.401
Anesthesia management				
TIVA	16 (45.7)	12 (48)	4 (40)	0.723
Tube advancement	27 (77)	18 (72)	9 (90)	0.390
DLT	11 (31)	4 (4.9)	0 (0.0)	0.120
Bronchus blocker	3 (9)	1 (4)	2 (20)	0.190
Devices during surgery	1 (0–2)	1 (0–2)	2 (0–2)	0.388
Tracheotomy				
Before surgery	7 (20)	4 (16)	3 (30)	0.761
During surgery	6 (17)	5 (20)	1 (10)	
After surgery	7 (20)	5 (20)	2 (20)	
Not performed	15 (43)	11 (44)	4 (40)	
Respiratory variables				
FiO_2_ 1.0, %	90 (75–100)	90 (55–100)	95 (80–100)	0.547
FiO_2_ other	0.62 (0.5–0.8)	0.6 (0.5–0.75)	0.65 (0.45–0.8)	0.961
p/f ratio before	141 (110–219)	133 (115–212)	157 (101–315)	0.860
p/f after	143 (111–153)	144 (133–154)	97 (80–236)	*0.042*
SaO_2_ lowest, %	76 (54–85)	84 (65–88)	54 (24–72)	*0.002*
etCO_2_ highest, mmHg	46 (41–59)	45 (40–59)	51 (42–60)	0.410
PIP highest, mmHg	25 (25–28)	25 (25–29)	25 (25–28)	0.621
PEEP highest, mmHg	10 (8–12)	10 (8–11)	10 (8–12)	0.509
Circulation				
SBP lowest, mmHg	74 (52–82)	80 (70–86)	40 (0–72)	*< 0.001*
Noradrenaline, μg kg min^−1^	0.18 (0.1–0.31)	0.10 (0.06–0.22)	0.22 (0.2–0.84)	^a^
Adrenaline	8 (22.9)	1 (4)	7 (70)	^a^
Dobutamine	9 (25.7)	5 (20)	4 (40)	0.398
Crystalloid, L	2 (1–2.5)	1.5 (1–2)	2.5 (1–4)	*0.009*
Transfusion	14 (40)	8 (32)	6 (60)	0.151
Blood loss, ml	450 (250–750)	350 (250–575)	725 (312–1150)	0.107
Lactate before, mmol/l	1.3 (0.8–2.6)	1.4 (1–2.6)	1.1 (0.7–2.4)	0.604
Lactate after, mmol/l	1.4 (1.1–3.1)	1.3 (0.9–2.9)	2.8 (1.5–5.6)	0.083
Urinary output, ml	50 (0–100)	50 (0–100)	25 (0–162)	0.734
Outcome				
Ventilator days	9 (4–18)	10 (4–25)	8 (3–14)	0.494
LOS ICU, days	10 (6–24)	11 (6–33)	9 (5–14)	0.118
All-cause 30-day mortality	16 (46)	7 (28)	9 (90)	*0.002*
Adjusted mortality	7 (20)	1 (4)	6 (60)	*0.001*

Data are medians (IQR) and counts (%); ^a^, comparisons not applicable due to categorization; *BMI* body mass index, *ASA* American Society of Anesthesiologists classification, *ICU* intensive care unit, *CPR* cardiopulmonary resuscitation, *SAPS II* simplified acute physiology score revision two, *SOFA* sequential organ failure assessment, *EMS* emergency medical service, *OLV* one-lung ventilation, *DLT* double lumen tube, *p/f* paO_2_/FiO_2_-ratio, *PIP* peak inspiratory pressure, *PEEP* positive end-expiratory pressure, *LOS* length of stay. *P* values below 0.05 are significant

### Perioperative management

**Fig. 4:**
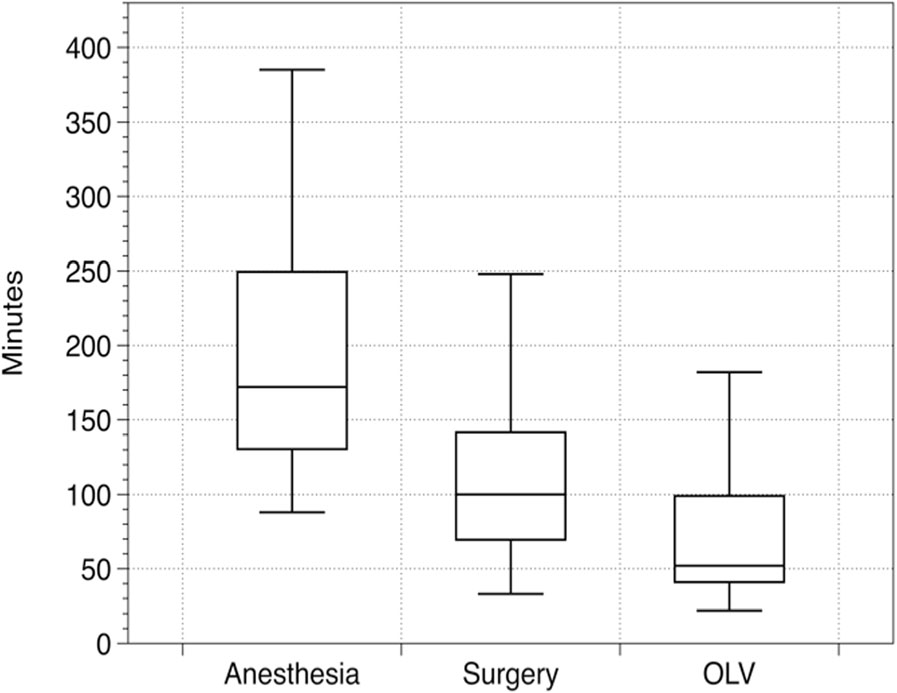
Procedure durations of surgical repair of iatrogenic tracheal ruptures. In the boxes, the dark horizontal line represents the median, and the box represents the 25th and 75th percentiles, the whiskers are the 5th and 95th percentiles. OLV: one-lung ventilation

Times from rupture to surgery and process times of anesthesia, surgery and OLV are presented in Table 1 and Fig. 4. Thirty-one patients (89%) were already under general anesthesia and mechanical ventilation prior to operating room admission. The remaining four patients (11%) underwent induction of general anesthesia in the operating room, one of whom required awake fiber-optic intubation due to oropharyngeal cancer and anticipated difficult airway. In all but three patients, arterial cannulation for invasive blood pressure monitoring and central venous catheterization (CVC) were already established in the ICU. Two patients received CVC after anesthesia induction, and one patient received it during surgery.

Propofol was used for anesthesia induction in 32 patients (91%), and nine patients (26%) underwent genuine propofol-based TIVA. Five patients received additional midazolam and midazolam/ketamine administration, and two patients received only midazolam. All patients received sufentanil for analgesia (seven patients received repeated bolus administrations, and 28 patients had continuous administration), and two patients were primarily induced with remifentanil (later replaced by sufentanil). Rocuronium was used for neuromuscular blockade in most patients (*n* = 32, 91%); two patients received pancuronium, and one patient received cis-atracurium due to liver failure. The anesthesia regimen included volatile and total intravenous anesthesia (TIVA) approaches, which were chosen at the discretion of the attending anesthetist (Table 1). Volatile anesthesia was applied in 19 patients (54%), and 16 of these patients received isoflurane, two received desflurane, and one received sevoflurane. Four patients who received volatile anesthesia received additional intravenous propofol and midazolam administration. Sixteen patients (46%) underwent TIVA, three of these patients secondary to volatile anesthesia approaches with the development of air leakage and exposure of anesthesia gases towards the operating team after OLV. All patients were transferred to the ICU under mechanical ventilation with propofol or midazolam sedation after surgery.

### Airway management and one-lung ventilation

Most patients (*n* = 27, 77%) underwent tube advancement into one main-stem bronchus for OLV. Two of these patients underwent additional approaches with bronchus blockers, and one patient received a bronchus blocker without previous tube advancement. Eleven patients (31%) had DLT placement, four of these placements were secondary to previous single-lumen tube advancements into one main bronchus without sufficient OLV conditions. All OLV approaches were monitored using fiber-optic bronchoscopy. Of the nine patients who had tracheostoma-related tracheal ruptures, four underwent orotracheal single-lumen tube intubation, another four underwent single-lumen intubation via tracheostomy, and one underwent double-lumen tracheal-cannula insertion for perioperative airway management.

The successful establishment of OLV required one tube exchange in eight patients (23%). Two exchanges were necessary in another 12 patients (34%), and three patients (9%) required three exchanges. Therefore, primary tube placement was successful in the remaining 12 patients (34%). Notably, only one of the 11 patients with DLT did not require tube exchanges. The respiratory variables before, during and after surgery are shown in Table 1.

OLV approaches were associated with intermittent circulatory arrest in four patients. OLV remained impossible in two of these patients. One patient was operated under ECMO support and weaned off the ECMO directly after surgery (ECMO duration 2 h). Another patient died in the operating room after prolonged cardiopulmonary resuscitation (CPR) efforts.

Iatrogenic tube cuff lesions and tube damage due to surgery were observed in five cases, most likely because of the very close proximity to the surgical field. In one case, the cuff was inadvertently sutured, and parts of the cuff were retrieved postoperatively via bronchoscopy. From the five patients with cuff lesion, a pharyngeal tamponade provided acceptable ventilation conditions in three patients. Airway leakage was minimal in the remaining two patients, and no further measures were necessary.

FiO_2_ during OLV varied considerably while 17 patients (49%) were ventilated with pure oxygen during the entire operation period. Eighteen patients had a median proportion of pure oxygen ventilation during 90% of the operating period, and the median FiO_2_ in the remaining time was 0.62. Patients with no and minor complications were comparable with patients who presented with major complications in the median FiO_2_, the p/f-ratio before surgery, and the highest etCO_2_, PIP and PEEP levels, and they exhibited significant differences in p/f-ratios after surgery (*p* = 0.042) and lowest SpO_2_ (*p* = 0.002) (Table 1).

Postoperatively, eleven patients underwent another tracheal tube exchange for DLT removal or for a tube with a larger diameter. Tracheotomy was performed prior to tracheal rupture in seven patients (being the cause of tracheal rupture in five cases), during tracheal surgery in six patients and after tracheal surgery in another six patients at the ICU after a median of three days (range 0–9 days). Fifteen patients did not undergo tracheotomy. All of the 34 patients who were postoperatively admitted to the ICU underwent frequent bronchoscopy re-evaluations.

### Circulation

Twenty-eight patients received continuous noradrenaline administration for circulation stabilization with the median highest noradrenaline dosage of 0.18 (0.1–0.31) μg kg min^− 1^. Nine patients received dobutamine, and another eight patients (including four who received dobutamine) received adrenaline. The median perioperative fluid administration was 2000 (1000-2500) ml using Ringers acetate, and six patients received additional 6% hydroxyethyl starch (500 ml each). Blood transfusion was required in fourteen patients (40%). Eight patients received only RBCs, four patients RBCs and FFP, and two patients received RBCs and platelet concentrates. The median total blood loss was 450 (250–750) ml, and the median total urinary output was 50 (0–100) ml. Thirteen patients (37%) had anuria during surgery, and four patients required cardiopulmonary resuscitation. Patients with major perioperative complications had significantly lower lowest SBP (*p* < 0.001), and received larger volumes of crystalloids (*p* = 0.009) compared to patients who had no and minor complications (Table 1).

### Surgery

Broad-spectrum antibiotic therapy was initiated prior to surgery in 31 patients (89%), primarily using carbapenem (55%). Twenty-six patients (74%) received additional antibiotic administration prior to skin incision in the operating room. Tracheal suturing was performed in all patients, and sutures were reinforced in 29 patients (83%) using artificial tissues (e.g., TachoSil®, TachoComb®, Sulmycin®) in nine patients and additional autologous tissue patches/flaps (e.g., pleura, pericardium, thymus, latissimus dorsi muscle and stylohyoid muscle) in 20 patients. Right-side chest tubes were inserted in all patients, and five patients received additional left side chest tubes due to pneumothorax. Surgical revisions were required in six patients (17%), of whom three underwent one revision and one underwent two revisions. Two patients with suture insufficiency after the first surgery could not be revised. One of these patients was too unstable for revision and died on postoperative day (POD) two, and the other patient died due to coincidental rupture of an aortic aneurysm during transfer to the operating room.

### Outcomes

The median length of stay in the ICU was 10 (6–24) days, including 9 (4–18) ventilator days (Table 1). With regard to preinjury morbidity, both patients with ASA I-II survived, but all-cause 30-day survival rates of patients with ASA III was 70% (7 of 10), ASA IV was 44% (7 of 16) and ASA V-VI was 0% (0 of 7) (Fig. 3). One patient was classified as ASA VI due to severe blunt trauma that included a nonsurvivable traumatic brain injury. This patient underwent prehospital CPR and difficult emergency intubation, which caused the tracheal rupture. After surgical repair and brain death protocol completion, the patient underwent organ donation of heart, liver and kidneys. Mortality related to tracheal surgery was 20% (*n* = 7 patients), including one patient who died in the operating room during surgery. All-cause 30-day mortality was 46% (*n* = 17). Eleven patients (65%) died from sepsis/multiorgan failure, and six (35%) patients died from acute cardiopulmonary decompensation (including the patient with aortic rupture on the way to surgical revision). Three more patients died after 30 days. One of these patients died due to fulminant mediastinitis on POD 31, and two patients died from cardiopulmonary decompensation on POD 41 and cerebral ischemia on POD 60. Patients with major complications had significantly higher all-cause 30-day mortality (*p* = 0.002) and adjusted mortality (*p* = 0.001) compared to patients who had no and minor complications.

## Discussion

### Key results

Thoracotomy for the surgical repair of iatrogenic tracheal ruptures is an emergency operation that is performed 2–3 times annually in our center, which is consistent with earlier data of our and other centers [3–7]. Because minor tracheal lesions are treated conservatively, and some lesions remain undetected, the true incidence of iatrogenic tracheal ruptures requiring surgical repairs is difficult to estimate [1–11]. Our results demonstrated the perioperative management and incidence of perioperative complications of a single center. The results of our study suggest that patients with iatrogenic tracheal ruptures present with considerable preinjury morbidity. Only a small number of our patients presented with ASA I or II, and most patients were already admitted to the ICU or underwent emergency tracheal intubation for cardiopulmonary resuscitation. We found that high preinjury morbidity may result in poor outcomes even with advanced healthcare resources, including specialized hospital infrastructures and medical expertise. Our results also indicate that major perioperative complications in these patients are associated with further deterioration of adjusted and all-cause 30-day mortality compared to patients with no and minor complications. Our all-cause 30-day mortality rate of 46% is consistent with the published literature, but other centers reported lower and higher mortality rates up to 70% [1, 3, 4, 8, 11]. However, detailed data of preinjury conditions are missing in most studies. Whether surgery should be avoided in highly morbid patients remains unanswered because potentially associated perioperative complications may be preventable or treatable. The decision to perform surgical repair should be made carefully depending on individual circumstances and keeping in mind that conservative approaches do not necessarily provide substantially better outcomes [2].

### Interpretation

We regard appropriate airway management and sufficient OLV as key measures for successful surgery. Tube advancement into one main-stem bronchus was the most common approach in our study, and DLT was used in only one-third of cases. This use is consistent with other centers that reported similar airway strategies [5]. Our data also reveal that intraoperative changes of airway devices were required frequently. In most cases, the tube was too large or too short and could not be advanced properly into one main-stem bronchus below the tracheal rupture and required replacement and insertion of a smaller and/or longer tube or DLT. OLV resulted in severe cardiopulmonary complications in almost one-third of our patients, including four patients who required CPR. Two patients did not tolerate OLV at all, and one patient received ECMO, which may be an emergency option for tracheal surgery when OLV is not possible [12, 13]. Transient hypoxemia in OLV is a common complication, and fiber-optic monitoring of tube position, adjustment of ventilation strategy and increasing the FiO_2_ prevent longer episodes [14, 15]. We observed a high proportion of pure oxygen ventilation despite considerably low levels of lowest SpO_2_. Although it may be presumed that patients with already impaired gas exchange may have had a higher probability of pure oxygen ventilation, proportions were comparable in both groups and thus did not influence outcome statistically. Compared to other studies of OLV in the setting of surgical repair of iatrogenic ruptures, our average PEEP levels were high [8]. The reason for the common practice of the application of low PIP and PEEP levels during tracheal surgery is to avoid shear stress in the respiratory system [5]. However, in prospective randomized clinical trials, lung-protective ventilation strategies during OLV did not improve gas exchange compared with conventional ventilation patterns [16]. Alternative airway approaches for surgical repair of iatrogenic tracheal ruptures include high-frequency jet ventilation (HFJV). Although it was not applied in our study collective, HFJV provides similar visualization of the operating field and may be a feasible alternative to DLT [17–19].

Although volatile anesthesia is generally assumed to exhibit lower shunt volumes and less inflammatory response in OLV, randomized controlled trials did not find relevant benefits compared to TIVA [20–23]. More than half of the patients in our study received volatile anesthesia, but volatile anesthesia was changed to TIVA in some patients because of gas exposure to the surgical team. Due to possible airway leakage during surgery under emergency conditions, the anesthetic approach should include a possibility to rapidly change from volatile anesthesia to TIVA.

We observed severe hypotensive episodes during OLV in our patients who required advanced fluid resuscitation, transfusion of blood products and administration of vasopressors and inotropic agents. This necessity reflects the high comorbidity of the study cohort and warrants arterial cannulation and CVC placement for this type of surgery. We did not perform perioperative advanced hemodynamic monitoring (i.e., cardiac output measurements) in our study cohort, and there is no evidence for its benefit in patients undergoing OLV (e.g., for prevention of over-infusion) [24, 25]. However, pre-existing conditions and underlying diseases must be taken into account and should be considered carefully during circulation management.

### Limitations

Because of the general limitations of retrospective observations, small sample sizes and a heterogeneous patient cohort, we must acknowledge the high mortality rates of our study, which may reflect the comorbidity of our patients and may not be comparable to other studies of healthier patients. Furthermore, the results may not be generalizable to the general population due to the single center study design. The treatment of our patients was not standardized apart from the surgical approach. Although only experienced anesthetists and surgeons were involved, different treatment approaches may have influenced individual patient outcome. Nevertheless, the present study provides new data on the emergency treatment of a rare patient cohort under real-life conditions.

## Conclusions

In our study, the comorbidity of patients undergoing thoracotomy for surgical repair of tracheal perforation was considerably high. The perioperative management required different individual approaches of airway management and circulation support. Patients with major perioperative complications were associated with higher postoperative morbidity and mortality. With regard to underlying diseases, we recommend close interdisciplinary communication to manage critical episodes appropriately and safely. Precautions for airway management should include early anticipation of alternative one-lung ventilation approaches to provide acceptable conditions for surgery.

## Data Availability

The datasets used and analyzed during the current study are available from the corresponding author upon reasonable request.

## Abbreviations

ARDS: acute respiratory distress syndrome
ASA: American Society of Anesthesiologists classification
CI: Confidence interval
CPR: Cardiopulmonary resuscitation
CT: Computed tomography
CVC: Central venous catheter
DLT: Double-lumen tube
ECLA: Extra-corporal lung assist
ECMO: Extra-corporal membrane oxygenation
ED: Emergency department
EMS: Emergency medical service
EtCO_2_: End-tidal carbon-dioxide level
FFP: Fresh frozen plasma
FiO_2_: Fraction of inspired oxygen
HFJV: High-frequency jet ventilation
ICD10: International classification of diseases revision 10
ICU: Intensive care unit
IQR: Interquartile range
LOS: Length of stay
OLV: One-lung ventilation
PaO_2_: Partial pressure of arterial oxygen
PEEP: Positive end-expiratory pressure
PIP: Peak inspiratory pressure
POD: Post-operative day
RBC: Red blood cells
SAPS 2: Simplified acute physiology score revision 2
SBP: Systolic blood pressure
SD: Standard deviation
SOFA: Sequential organ failure assessment
SpO_2_: Peripheral oxygen saturation
TIVA: Total intravenous anesthesia
